# A parasitological evaluation of edible insects and their role in the transmission of parasitic diseases to humans and animals

**DOI:** 10.1371/journal.pone.0219303

**Published:** 2019-07-08

**Authors:** Remigiusz Gałęcki, Rajmund Sokół

**Affiliations:** 1 Department of Veterinary Prevention and Feed Hygiene, Faculty of Veterinary Medicine, University of Warmia and Mazury, Olsztyn, Poland; 2 Department of Parasitology and Invasive Diseases, Faculty of Veterinary Medicine, University of Warmia and Mazury, Olsztyn, Poland; Universidade Federal do Rio de Janeiro, BRAZIL

## Abstract

From 1 January 2018 came into force Regulation (EU) 2015/2238 of the European Parliament and of the Council of 25 November 2015, introducing the concept of “novel foods”, including insects and their parts. One of the most commonly used species of insects are: mealworms (*Tenebrio molitor*), house crickets (*Acheta domesticus*), cockroaches (Blattodea) and migratory locusts (*Locusta migrans*). In this context, the unfathomable issue is the role of edible insects in transmitting parasitic diseases that can cause significant losses in their breeding and may pose a threat to humans and animals. The aim of this study was to identify and evaluate the developmental forms of parasites colonizing edible insects in household farms and pet stores in Central Europe and to determine the potential risk of parasitic infections for humans and animals. The experimental material comprised samples of live insects (imagines) from 300 household farms and pet stores, including 75 mealworm farms, 75 house cricket farms, 75 Madagascar hissing cockroach farms and 75 migrating locust farms. Parasites were detected in 244 (81.33%) out of 300 (100%) examined insect farms. In 206 (68.67%) of the cases, the identified parasites were pathogenic for insects only; in 106 (35.33%) cases, parasites were potentially parasitic for animals; and in 91 (30.33%) cases, parasites were potentially pathogenic for humans. Edible insects are an underestimated reservoir of human and animal parasites. Our research indicates the important role of these insects in the epidemiology of parasites pathogenic to vertebrates. Conducted parasitological examination suggests that edible insects may be the most important parasite vector for domestic insectivorous animals. According to our studies the future research should focus on the need for constant monitoring of studied insect farms for pathogens, thus increasing food and feed safety.

## Introduction

The growing demand for easily digestible and nutritious foods has contributed to the emergence of new food sources in agricultural processing. Edible insects are one such category of under-utilized foods with a high nutritional value [1]. Insects are farmed for direct consumption and for use in the production of foods and feeds [2]. The concept of “novel foods”, including insects and their parts, has been introduced by Regulation (EU) 2015/2238 of the European Parliament and of the Council of 25 November 2015 on novel foods, which came into force on 1 January 2018. The growing popularity of exotic pets has also increased the demand for novel foods. However, edible insects are often infected by pathogens and parasites which cause significant production losses [3]. These pathogens also pose an indirect threat for humans, livestock and exotic animals. The majority of insect farming enterprises in the world are household businesses, and in Europe edible insects are rarely produced on a large scale. In European Union, entomophagy is rare, and it is regarded as a cultural taboo [4]. More than 1900 species of insects are considered to be edible. The most popular edible insects include mealworms (*Tenebrio molitor*) [5], house crickets (*Acheta domesticus*) [4], cockroaches (Blattodea) [6] and migratory locusts (*Locusta migrans*) [4].

Mealworms are beetles of the family Tenebrionidae. Adult beetles are generally 13-20 mm in length, and larvae have a length of around 30 mm. During their short life cycle of 1-2 months, females lay around 500 eggs. One of the largest mealworm suppliers in the world is HaoCheng Mealworm Inc. which produces 50 tons of live insects per month and exports 200,000 tons of dried insects per year [7]. Mealworms are used in human and animal nutrition, and they are a popular food source for exotic pets, including reptiles and insectivores. The nutritional value of mealworm larvae is comparable to that of meat and chicken eggs [8]. Mealworms are easy to store and transport. They are abundant in highly available nutrients and are regarded as a highly promising source of feed in poultry and fish breeding. Mealworms can also be administered to pets and livestock [4]. The popularity of mealworms consumption by humans is on the rise especially in Europe. Mealworms effectively degrade biological waste and polystyrene foam [9]. The most common mealworm parasites include *Gregarine* spp., *Hymenolepis diminuta* and mites of the family Acaridae. Mealworms are model insects in parasitological research [10–12].

The house cricket (*A. domesticus*) has a length of up to 19 mm, and its life cycle spans 2-3 months. It is a source of food for reptiles, amphibians and captive bred arachnids, including spiders of the family Theraphosidae. House crickets are consumed by humans in powdered form or as protein extracts [13, 14]. Whole crickets are consumed directly in Thailand [1]. These insects are frequently infested by *Nosema* spp., *Gregarine* spp. and *Steinernema* spp.

Cockroaches of the order Blattodea include the German cockroach (*Blattella germanica*), American cockroach (*Periplaneta americana*), Cuban burrowing cockroach (*Byrsotria fumigata*), Madagascar hissing cockroach (*Gromphadorhina portentosa*), speckled cockroach (*Nauphoeta cinerea*), Turkestan cockroach (*Shelfordella lateralis*) and oriental cockroach (*Blatta orientalis*). Cockroaches can live for up to 12 months, and the largest individuals reach up to 8 cm in length. Cockroaches are increasingly popular in human nutrition, and they are a part of the local cuisine in various regions of the world [15].

Migratory locusts are members of the family Acrididae, order Orthoptera. Insects have up to 9 cm in length and live for up to 3 months. Locusts are consumed by amphibians, reptiles and humans, mainly in Africa and Asia. Locusts contain up to 28% protein and 11.5% fat, including up to 54% of unsaturated fats [16]. *Nosema* spp. and *Gregarine* spp. are the most prevalent locust parasites [17].

The aim of this study was to identify and evaluate the developmental forms of parasites colonizing edible insects in household farms and pet stores in Central Europe and to determine the potential risk of parasitic infections for humans and animals.

## Materials and methods

### Materials

The experimental material comprised samples of live insects (imagines) from 300 household farms and pet stores, including 75 mealworm farms, 75 house cricket farms, 75 Madagascar hissing cockroach farms and 75 migrating locust farms from Czechia, Germany, Lithuania, Poland, Slovakia and Ukraine. Owners/breeders of household farms and cultures from pet stores gave permission for the study to be conducted on their insect farms. The studies were carried out in the years 2015-2018. Up to 3 farms were tested from a single location (eg. city). Farm stock was purchased from suppliers in Europe, Asia and Africa. Forty insects were obtained from every mealworm and cricket farm, and they were pooled into 4 samples of 10 insects each. Ten insects were sampled from every cockroach and locust farm, and they were analyzed individually.

### Methodology

Insects were immobilized by inducing chill coma at a temperature of -30°C for 20 minutes. Hibernation was considered effective when legs, mandibles and antennae did not respond to tactile stimuli. Hibernating insects were decapitated and dissected to harvest digestive tracts. Digestive tracts were ground in a sieve and examined by Fulleborn’s floatation method with Darling’s solution (50% saturated NaCl solution and 50% glycerol). The samples were centrifuged at 3500 x for 5 minutes. Three specimens were obtained from every sample, and they were examined under a light microscope (at 200x, 400x and 1000x magnification). The remaining body parts were examined for the presence of parasitic larvae under the Leica M165C stereoscopic microscope (at 7.2x-120x magnification) The remaining body parts were analyzed according the method proposed by Kirkor with some modifications, by grinding body parts in a mortar with a corresponding amount of water and 0.5 ml of ether. The resulting suspensions were filtered into test tubes to separate large particles and were centrifuged at 3500x for 5 minutes. After loosening the debris plug, the top three layers of suspension were discarded. Three specimens were obtained, and they were analyzed according to the procedure described above. Parasites were identified to genus/species level based on morphological and morphometric parameters with the use of an Olympus image acquisition system and Leica Application Suite program based on the reference sources in Pubmed [18–36]. Parasites were identified to species level by Ziehl-Neelsen staining [37]. The owners of farms where human parasites were detected were advised to eliminate their stock. Farm owners were surveyed with the use of a questionnaire to elicit information about the origin of insects (to determine whether the stock was supplemented with insects from other farms, whether the farm was a closed habitat, whether stock was obtained only from Europe, or also from Asia/Africa), insect nutrition (whether insects were fed specialized feeds, fresh products, kitchen discards or locally collected sources of feed), contact with other animals or animal feces.

### Statistical analysis

The prevalence of parasitic species was determined for every insect species. The data were tested for normal distribution with the Kolmogorov-Smirnov test. The assumptions of linearity and normality were tested before statistical analysis. Linearity was analyzed based on two-dimensional distribution of the evaluated variables with the use of histograms and normal probability plots of the residuals. The significance of the correlations between insect species and questionnaire data was analyzed in a logistic regression model, where the dependent variable was dichotomous (0 or 1, presence/absence of parasites) and the independent variables were: origin of insects (insects purchased in Europe only/insects imported from Asia and Africa), Insect stock rotation system (insects from the evaluated farm only- close rotation/the farm was supplemented with insects from other farms- open rotation), nutrition (insects fed only fresh products or specialized feeds/insects fed kitchen discards) and direct/indirect contact with animals (yes/no). The correlations between the identified parasites were analyzed with the use of Yule’s Q and Cramer’s V, subject to the number of the evaluated variables. The examined associations were weak when the value of V/Q approximated 0, and the correlations were stronger when V/Q approximated +1/-1. The results were processed statistically in the Statistica 13.1 program with a StatSoft medical application.

## Results

### Prevalence

Parasitic developmental forms were detected in 244 (81.33%) out of 300 (100%) examined insect farms. In 206 (68.67%) of the cases, the identified parasites were pathogenic for insects only; in 106 (35.33%) cases, parasites were potentially parasitic for animals; and in 91 (30.33%) cases, parasites were potentially pathogenic for humans. *Nosema* spp. spores were detected in 27 (36.00%) cricket farms and 35 (46.67%) locust farms. The presence of *Cryptosporidium* spp. was observed in 12 (16%) mealworm farms, 5 (6.67%) cricket farms, 13 (17.33%) cockroach farms and 4 (5.33%) locust farms. Forty-four (58.67%) mealworm farms, 30 (40.00%) cricket farms, 57 (76%) cockroach farms and 51 (68.00%) locust farms were infested with *Gregarine* spp., including *Steganorhynchus dunwodyii*, *Hoplorhynchus acanthatholius*, *Blabericola haasi*, *Gregarina blattarum*, *G. niphadrones*, *Gregarina cuneata* and *Gregarina polymorpha*. *Isospora* spp. were detected in 7 (9.33%) mealworm farms, 4 (5.33%) cricket farms, 9 (12.00%) cockroach farms and 8 (10.67%) locust farms. Eleven (14.67%) mealworm farms, 13 (17.33%) cockroach farms and 9 (12.00%) locust farms were infested with *Balantidium* spp. including *B. coli* and *B. blattarum*. The presence of *Entamoeba* spp., including *E. coli*, *E. dispar*, *E. hartmanii* and *E. histolytica*, was determined in 9 (12%) mealworm farms, 14 (18.67%) cockroach farms and 4 (5.33%) locust farms. Seventeen (22.67%) cockroach farms were colonized by *Nyctotherus* spp., including *N. ovalis* and *N. periplanetae*. Tapeworm cysticercoids, including *Hymenolepis nana*, *H. diminuta* and *Raillietina* spp., were detected in 9 (12%) mealworm farms, 3 (4%) cricket farms, 4 (5.33%) cockroach farms and 3 (4.00%) locust farms. Nematodes of the order Gordiidea colonized 6 (8.00%) cricket and locust farms. *Hammerschmidtiella diesigni* was detected in 35 (46.67%) cockroach farms. *Steinernema* spp. was identified in 22 (29.33%) cricket farms, and *Pharyngodon* spp.—in 14 (18.67%) locust farms. The presence of *Physaloptera* spp. was observed in 4 (5.4%) mealworm farms, 2 (2.67%) cricket farms, 9 (12.00%) cockroach farms and 7 (9.33%) locust farms. Five (6.67%) mealworm farms and 7 (9.33%) cockroach farms were infested with Spiruroidea. Thelastomidae spp. was detected in 10 (13.33%) cricket and locust farms. *Thelastoma* spp. was identified in 58 (77.33%) cockroach farms. Acanthocephala were observed in 2 (2.67%) mealworm farms and 3 (4.00%) cockroach farms. Two (2.67%) cockroach farms were infested with Pentastomida. The presence of Acaridae, including house dust mites, was observed in 35 (46.67%) mealworm farms, 15 (20.00%) cockroach farms and 7 (9.33%) locust farms. In the group of samples collected from mealworm farms, *Cryptosporidium* spp. were noted in 37 (12.33%) samples, *Gregarine* spp. were detected in 99 (33.00%) samples, *Isospora* spp.—in 12 (4%) samples, *Entamoeba* spp.—in 12 (4.00%) samples, *Balantidium* spp.—in 14 (4.67%) samples, cysticercoids—in 18 (6.00%) samples, *Pharyngodon* spp.—in 10 (3%) of samples, *Physaloptera* spp.—in 15 (5.00%) samples, *Spiruroidea*—in 6 (2.00%) samples, Acanthocephala spp.—in 2 (0.67%), and Acaridae in 80 (26.67%) samples. In the group of samples collected from cricket farms, *Nosema* spp. were identified in 74 (24.67%) samples, *Cryptosporidium* spp.—in 5 (1.67%) samples *Isospora* spp.—in 8 (2.67%) samples, *Gregarine* spp.—in 72 (24.00%) samples, cysticercoids—in 4 (1.33%) samples, *Physaloptera* spp.—in 4 (1.33%) samples, *Steinernema* spp.—in 11 (3.67%) samples, and nematodes of the order Gordiidea—in 19 (6.33%) samples. In the group of samples obtained from cockroach farms, the presence of *Cryptosporidium* spp. was determined in 89 (11.87%) samples, *Gregarine* spp.—in 236 (31.47%) samples, *Isospora* spp.—in 16 (2.13%) samples, *Nyctotherus* spp.—in 57 (7.60%) samples, *Entamoeba* spp.—in 34 (4.53%) samples, *Balantidium* spp.—in 35 (4.67%) samples, cysticercoids—in 4 (0.53%) samples, *Pharyngodon* spp.—in 20 (2.67%) samples, *Physaloptera* spp.—in 23 (3.07%) samples, Spiruroidea—in 14 (1.87%) samples, *Thelastoma* spp.—in 270 (36.00%) samples, *H. diesigni*—in 143 (19.07%) samples, Acanthocephala spp.—in 5 (0.67%) samples, Pentastomida spp.—in 5 (0.67%) samples, and Acaridae—in 29 (3.87%) samples. The following parasites were identified in locust farms: *Nosema* spp.—in 125 (16.67%) samples, *Cryptosporidium* spp.—in 13 (1.73%) samples, *Gregarine* spp.—in 180 (24.00%) samples, *Isospora* spp.—in 15 (2.00%) samples, *Entamoeba* spp. in 9 (1.20%) samples, *Balantidium* spp.—in 14 (1.87%) samples, cysticercoids—in 15 (2.00%) samples, *Physaloptera* spp.—in 17 (2.27%) samples, *Steinernema* spp.—in 31 (4.13%) samples, nematodes of the order Gordiidea—in 7 (0.93%) samples, and Acaridae—in 31 (4.13%) samples. Detailed results of the parasitological examination have been placed in Table 1.

**Table 1:** Type / Species and developmental forms of parasites found in the examined insects in the examined collective / individual samples depending on the place of detection.

Parasite (developmental forms)	Mealworm beetle		House cricket		Madagascar hissing cockroach		Migrating locust	
	g.t.	r.b.	g.t.	r.b.	g.t.	r.b.	g.t.	r.b.
*Nosema* spp. (spores)	-	-	74	-	-	-	125	-
*Cryptosporidium* spp. (oocysts)	31	10	5	2	57	35	13	4
*Gregarine* spp. (oocysts, sporozoites)	99	-	72	-	236	-	180	-
*Isospora* spp. (oocysts)	3	12	1	8	6	16	1	15
*Balantidium* spp. (amoeba, cysts)	1	14	-	-	29	8	5	14
*Entamoeba* spp. (amoeba, cysts)	3	11	-	-	30	7	1	9
*Nyctotherus* spp. (amoeba, cysts)	-	-	-	-	57	2	-	-
Cestoda (eggs, cysticercoids)	8	22	-	4	3	4	2	15
*Gordiidae* spp (cysts, juveniles)	-	-	-	19	-	-	16	-
*H. diesigni*(adult forms, eggs)	-	-	-	-	143	-	-	-
*Pharyngodon* spp. (L3 larvae)	-	13	-	-	-	22	-	-
*Physaloptera* spp. (L3 larvae)	-	19	-	4	-	42	-	17
Spiruroidea (L3 larvae)	-	8	-	-	-	14	-	-
Thelastomatidae (adult forms, eggs)	-	-	47	-	-	-	31	-
*Steinernema* spp. (adult forms)	-	-	-	11	-	-	-	17
*Thelastoma* spp. (adult forms, eggs)	-	-	-	-	270	-	-	-
Acanthocephala spp. (cystacanths)	-	2	-	-	-	5	-	-
Pentastomida (nymphs)	-	-	-	-	-	5	-	-
Acaridae (eggs, nymphs, adult forms)	4	80	-	-	2	29	1	31

g.t.—gastrointestinal tract; r.b.—rest of the body

### Probability of parasite occurrence

The risk of Cestoda, Acanthocephala and Acaridae infections was significantly higher in insects imported from Africa and Asia than in insects purchased from European suppliers. Farms whose stock was supplemented with insects from other farms were more frequently colonized by *Nosema* spp., *Isospora* spp., *Cryptosporidium* spp., *Entamoeba* spp., Cestoda, *Pharyngodon* spp., *Gordius* spp., *Physaloptera* spp., *Thelastoma* spp. and *H. diesigni* than closed farms. The risk of infection with *Cryprosporidium* spp., *Gregarine* spp, *Balantidium* spp, *Entamoeba* spp., *Steinernema* spp., Gordiidea, *H. diesigni* and Acaridae was higher in insects fed kitchen discards and locally collected feed sources than insects fed only fresh products or specialized feeds. Insects that came into direct or indirect contact with animals were at higher risk of exposure to *Isospora* spp., *Cryptosporidium* spp., Cestoda, *Pharyngodon* spp., *Physaloptera* spp., *Thelastoma* spp. and *H. diesigni*, but at lower risk of exposure to *Nyctotherus* spp. The statistical significant results of logistic regression were placed in Table 2.

**Table 2:** Logistic regression model, showing statistically significant relationships between the parasite species and the origin of insects, insect stock rotation system, type of feeding and contact with animals.

*Nosema* spp.	rotation	<0.000001	2.28	0.18	33.99	<0.000001	2.90	2.03-4.14
*Isospora* spp.	rotation	0.000043	28.88	0.31	10.45	0.0012	2.74	1.49-5.06
	animals		12.03	0.32	3.95	0.047	1.87	1.01-3.48
*Cryprosporidium* spp.	rotation	0.00001	14.54	0.22	15.53	0.00002	5.11	1.03-14.65
	feeding		17.76	0.19	19.22	0.0013	10.21	0.81-6.45
	animals		4.03	0.34	7.81	0.001	3.62	1.95-12.83
*Gregarine* spp.	feeding	0.000001	11.85	0.11	21.40	0.000004	1.65	1.33-2.04
*Nyctotherus* spp.	animals	0.020	49.02	0.29	8.29	0.004	0.44	0.25-0.77
*Balantidium* spp.	feeding	0.000001	6.43	0.32	15.63	0.000072	3.52	1.69-6.57
*Entamoeba* spp.	rotation	0.000022	4.50	0.54	5.78	0.016	0.27	0.095-0.79
	feeding		3.58	0.34	11.03	0.000098	3.13	1.60-6.13
Cestoda	origin	0.000064	11.66	1.06	4.71	0.03	10.07	1.25-81.05
	rotation		8.38	4.46	4.59	0.035	2.92	1.08-7.92
	animals		2.48	1.50	6.46	0.011	18.54	1.95-177.14
*Pharyngodon* spp.	rotation	0.000001	8.24	0.63	4.25	0.040	0.27	0.078-0.93
	animals		11.21	0.73	14.10	0.00017	15.73	3.73-66.31
*Steinernema* spp.	feeding	0.047	15.26	0.28	5.46	0.019	1.94	1.11-3.39
Gordiidae	rotation	0.000001	1.44	0.41	5.87	0.02	2.69	1.21-5.97
	feeding		4.89	1.03	18.67	0.000016	87.54	11.51-665.54
*Physaloptera* spp.	rotation	0.000001	12.28	0.36	8.62	0.0033	0.35	0.17-0.70
	animals		7.45	0.29	28.18	<0.000001	4.75	2.67-8.45
*Thelastoma* spp.	rotation	0.00087	33.09	0.19	4.61	0.031	1.51	1.04- 2.21
	animals		9.44	0.16	4.89	0.0002	1.26	1.26-2.43
*Hammerschmidtiella diesigni*	rotation	<0.000001	11.15	0.22	14.09	0.00017	2.32	1.49-3.59
	feeding		7.64	0.22	12.41	0.00042	2.18	1.41-3.73
	animals		5.82	0.20	7.47	0.0062	1.75	1.17-2.61
Acanthocephala	origin	0.00001	14.23	0.55	5.11	0.02	9.11	1.67-73.01
Acaridae	origin	0.000001	5.89	0.20	13.72	0.00021	2.08	1.41-3.06
	feeding		7.43	0.20	11.52	0.00069	1.99	1.34-2.96

*X*^2^—Chi-square test; *W*—Wald statistic; *95% CI*—95% Confidence Interval; Origin: 0—specimens in the breeding came from Europe, 1- breeding individuals imported from Asia or Africa; Rotation: 0—individuals from farms with closed animal rotation, 1—individuals from farms with open animal rotation; Feeding: 0—insects fed with fresh products or food, 1—insects fed with waste; Animals: 0—no contact with animals, 1—contact with animals. *H.diesigni*—*Hammerschmidtiella diesigni*.

### Coinvasions

Significant correlations were observed between the presence of *Nosema* spp. and *Isospora* spp. (V = 0.75), *Gregarine* spp. (Q = −0.27) *Steinernema* spp. (Q = 0.42) and Gordiidae spp (Q = 0.45). The presence of *Isospora* spp. was also significantly correlated with *Gregarine* spp. (Q = -0.22), cestoda (Q = 0.63), Gordiidae spp. (Q = 0.73) *Thelastoma* spp. (Q = 0.96). The occurrence of *Nyctotherus* spp. was correlated with Spiruroidea (Q = 0.55), *Thelastoma* spp. (Q = 0.52) and *H. diesigni* (Q = 0.18). Correlations were observed between *Gregarine* spp. and *Hymenolepis diminuta* (Q = 0.48), *Pharyngodon* spp. (Q = 0.30), Steinernema spp. (Q = 0.33), *Physaloptera* spp. (Q = 0.32), Spiruroidea. (Q = 0.44), *Thelastoma* spp. (Q = 0.51), *H. diesigni* (Q = 0.31) and Acanthocephala (Q = 0.44). The presence of *Cryptosporidium* spp. was significantly correlated with *Balantidium* spp. (Q = 0.21), *Entamoeba* spp. (Q = 0.33), *Nyctotherus* spp. (Q = −0.41), *H. diesigni* (Q = 0.49) and Acaridae (Q = 0.17).

## Discussion

Due to the lack of registration obligation, we are currently unable to estimate the exact number of such farms in the surveyed area. The number of farms obtained for the experiment resulted from an indicatively calculated minimum number of samples. To get the most reliable results from a single location (eg. city), we tested up to 3 farms. The selection of insect species for research resulted from the dissemination of these animals among breeders. In case we have shown that insects came from the same supplier, we did not continue further research.

Survey questions regarding the tested insect farms are related to the observed activities practiced by breeders. Breeders wanting to set up or enlarge their farms often order insects from the countries of origin or from places where the import of such food is cheaper than from Europe. In our opinion, such a phenomenon is a big threat due to the fact that there may be a risk of catching animals from the environment, and thus introducing new parasites, both pathogenic for insects as well as humans and animals. Some amateur breeders are not interested in the quality of feed introduced into the farm. They obtain insect feed from the environment (green fodder, wild fruit trees) or use leftovers from feeding other animals. Edible insects may also have direct or indirect contact with animals. Among the practices we can include re-depositing insects to farms after the animal has not eaten them. These insects moving around the animal habitat (eg. terrariums) can mechanically introduce potential pathogens specific to animals.

During the research in individual farms, we observed unethical practices of individual breeders, such as feeding insects with animal feces from a pet shop, feeding insects with corpses of smaller animals, or feeding insects with moldy food and even raw meat. These practices significantly reduce the quality of the final product and undermine the microbiological / parasitological safety of such food. Currently, however, there are no regulations regarding zoohygienic conditions and the welfare of these animals as potential animals for food. Although the research was conducted on amateur insect farms, most were not found to be seriously flawed. Breeding of edible insects carried out in places not intended for this purpose (houses) can lead to additional danger for humans. In the course of the study, we recorded individual cases of spreading insects from farms, which resulted in rooms infestation, eg. by cockroaches or crickets. Another example is the possibility of transmission of parasites such as *Cryptosporidium* spp. on human aerogenically, therefore if the farms are unprotected well or there is a lack of hygiene in contact with insects, such invasions may occur.

### Parasites pathogenic for insects

The analyzed farm samples were colonized by developmental forms of parasites that are specific to insects, including *Nosema* spp, *Gregarine* spp., *Nyctotherus* spp., *Steinernema* spp., Gordiidae, *H. diesigni*, Thelastomidae, and *Thelastoma* spp. These pathogens constitute the most prevalent parasitic flora, and massive infections can compromise insect health and decrease farm profits [38, 39]. According to Van der Geest et al. [40] and Johny et al. [41], the above pathogens have been implicated as pseudo-parasites of humans and animals. However, the impact of insect-specific parasites on humans has not yet been fully elucidated. Pong et al. [42] argued that *Gregarine* spp., a parasite specific to cockroaches, could cause asthma in humans. The results of the survey conducted in our study indicate that insect farming can increase the human exposure to pathogens and allergens [43, 44].

Nosemosis is a disease caused by microsporidian parasites, and it can compromise the health of crickets and locusts. However, nosema parasites also control cricket and locust populations in the natural environment [45–47]. Lange and Wysiecki [48] found that *Nosema locustae* can be transmitted by wild locusts to a distance of up to 75 km. This parasite is also readily transmitted between individual insects, which can contribute to the spread of infections in insect farms. Johnson and Pavlikova [49] reported a linear correlation between the number of *Nosema* spp. spores in locusts and a decrease in dry matter consumption. The results of our study indicate that *Nosema* spp. infections can decrease profits in insect farming.

*Gregarine* spp. are parasitic protists which colonize the digestive tracts and body cavities of invertebrates. According to Kudo [50], Gregarines are non-pathogenic commensal microorganisms, but recent research indicates that these protists are pathogenic for insects. These microorganisms utilize the nutrients ingested by the host, compromise the host’s immune function and damage the walls of host cells [41]. Massive infestations can lead to intestinal blockage in insects [38]. Lopes and Alves [39] found that cockroaches infected with *Gregarine* spp. were characterized by swollen abdomens, slower movement, darkened bodies and putrid smell indicative of septicemia. Gregarines were also found to compromise reproduction, shorten the life cycle and increase mortality in insects [38, 51, 52]. A study of dragonflies revealed that *Gregarine* spp. can decrease fat content and muscle strength in insects [52]. Johny et al. [41] demonstrated that metronidazole and griseofluvin can decrease *Gregarine* spp. counts in insects. The results presented by Johny et al. [41] can be used to develop parasite management strategies and minimize the negative effects of *Gregarine* spp. in insect farms. Lopes and Alves [39] demonstrated that cockroaches infected with *Gregarine* spp. were more susceptible to *Metarhizium anisopliae* and triflumuron, which could imply that diseased insects are more sensitive to other pathogens. A review of the literature suggests that *Gregarine* spp. can negatively affect the health of farmed insects [38, 39, 41, 51, 52].

*Nyctotherus* spp. is a parasite or an endosymbiont which colonizes the intestinal system of insects. Gijzen et al. [53] found a strong correlation between the size of the *N. ovalis* population and carboxymethyl-cellulase and filter paper digesting activity in cockroach intestines, which was correlated with those insects’ ability to produce methane. The results of our study indicate that the ciliate *N. ovalis* should be consider as commensal microflora of cockroach gastrointestinal tract. *Nyctotherus* spp. were less likely to be detected in insects that had previous contact with animals. The above could imply that insects whose digestive tracts are colonized by these parasites are more readily consumed by animals. *Nyctotherus ovalis* is rarely pathogenic for animals. Satbige et al. [54] reported on two turtles where *N. ovalis* infection caused diarrhea, dehydration and weight loss.

Gordiidae, also known as horsehair worms, are parasitic nematodes with a length of up to 1.5 m that colonize invertebrates. When consumed by insects, parasitic larvae penetrate the intestinal wall and are enveloped by protective cysts inside the gut. *Gordius* spp. are generally specific to insects, but these nematodes have also been detected in humans and animals. Several cases of parasitism and pseudo-parasitism by gordiid worms from various locations, including France, Italy, Bavaria, Dalmatia, East Africa, Southeast Africa, West Africa, Transvaal, Chile, United States and Canada, were described in the literature [55]. Horsehair worms were also identified in vomit and feces [56, 57]. However, none of the described parasitic invasions were pathogenic for humans. In the present study, parasites were detected in insects fed kitchen discards or locally collected food sources.

*Hammerschmidtiella diesigni*, *Thelastoma* spp. and Thelastomatidae are nematodes specific to invertebrates. Nematodes colonizing insect digestive tracts are generally regarded as commensal organisms. Taylor [58] demonstrated that *Leidynema* spp. exerted a negative effect on hindgut tissues in insects. Similarly to the pathogens identified in our study, *Leidynema* spp. belong to the family Thelastomatidae. Capinera [59] demonstrated that these nematodes can increase mortality in cockroach farms. In our study, insects colonized by *H. diesigni* and *Thelastoma* spp. were characterized by lower fat tissue content. McCallister [60] reported a higher prevalence of *H. diesigni* and *T. bulhoes* nematodes in female and adult cockroaches, but did not observe significant variations in differential hemocyte counts or hemolymph specific gravity [60].

*Steinernema* spp. is an entomopathogenic nematode whose pathogenicity is linked with the presence of symbiotic bacteria in parasitic intestines. These nematodes are used in agriculture as biological control agents of crop pests [61], which can promote the spread of infection to other insects. In our study, insects infected with *Steinernema* spp. were probably fed plants contaminated with parasite eggs.

### Parasites pathogenic for humans and animals

*Cryptosporidium* spp. are parasites that colonize the digestive and respiratory tracts of more than 280 vertebrate and invertebrate species. They have been linked with many animal diseases involving chronic diarrhea [62–64]. According to the literature, insects can serve as mechanical vectors of these parasites. Flies may be vectors of *Cryptosporidium* spp. that carry oocysts in their digestive tract and contaminate food [65, 66]. Earth-boring dung beetles [67] and cockroaches [68] can also act as mechanical vectors of these parasites in the environment. However, the prevalence of *Cryptosporidium* spp. in edible insects has not been documented in the literature. In our study, *Cryptosporidium* spp. were detected in the digestive tract and other body parts of all evaluated insect species. In our opinion, insects are an underestimated vector of *Cryptosporidium* spp., and they significantly contribute to the spread of these parasites.

*Isospora* spp. are cosmopolitan protozoa of the subclass Coccidia which cause an intestinal disease known as isosporiasis. These parasites pose a threat for both humans (in particular immunosuppressed individuals) and animals. The host becomes infected by ingesting oocytes, and the infection presents mainly with gastrointestinal symptoms (watery diarrhea). According to the literature, cockroaches, houseflies and dung beetles can act as mechanical vectors of Isospora spp. [69, 70]. In our study, insect farms were contaminated with this protozoan, which could be the cause of recurring coccidiosis in insectivores. *Isospora* spp. were detected on the surface of the body and in the intestinal tracts of insects. In our opinion, the presence of *Isospora* spp. in edible insects results from poor hygiene standards in insect farms.

*Balantidium* spp. are cosmopolitan protozoans of the class Ciliata. Some species constitute commensal flora of animals, but they can also cause a disease known as balantidiasis. According to the literature, these protozoans are ubiquitous in synanthropic insects [68, 71]. In some insects, *Balantidium* spp. is considered a part of normal gut flora, and it can participate in digestive processes [72]. Insects can be vectors of *Balantidium* spp. pathogenic for humans and animals [73]. In our study, potentially pathogenic ciliates were detected even in insect farms with closed habitats.

*Entamoeba* spp. are amoeboids of the taxonomic group Amoebozoa which are internal or commensal parasites in humans and animals. The majority of *Entamoeba* spp., including *E. coli*, *E. dispar* and *E. hartmanni*, identified in our study belong to non-pathogenic commensal gut microflora. However, pathogenic *E. histolytica* [74], and *E. invadens* were also detected in the presented study. *Entamoeba histolytica* can cause dysentery in humans and animals, whereas *E. invadens* is particularly dangerous for insectivorous animals such as reptiles and amphibians. Other authors demonstrated that *E. histolytica* is transmitted by insects in the natural environment [68, 75].

Cestoda colonize insects as intermediate hosts. Cysticercoids, the larval stage of tapeworms such as *Dipylidium caninum*, *Hymenolepis diminuta*, *H. nana*, *H. microstoma*, *H. citelli*, *Monobothrium ulmeri* and *Raillietina cesticillus*, have been identified in insects [76–78]. Insects have developed immune mechanisms that inhibit the development of these parasites [78, 79]. Tapeworms can induce behavioral changes in insects, such as significant decrease in activity and photophobic behavior [80]. Behavioral changes can prompt definitive hosts to consume insects containing cysticercoids. Our study demonstrated that insect farms which are exposed to contact with animals and farms which are supplemented with insects from external sources are at greater risk of tapeworm infection. Similar results were reported in studies of synanthropic insects [81, 82]. In our study, both cysticercoids and eggs were detected, which suggests that farms can be continuously exposed to sources of infection. However, the correlations between edible insects and the prevalence of taeniasis in humans and animals have never been investigated in detail. Temperature has been shown to significantly influence the development of tapeworm larvae in insects [83, 84]. In our opinion, the maintenance of lower temperature in insect farms could substantially decrease the reproductive success of tapeworms, and edible insects could be thermally processed before consumption to minimize the risk of tapeworm infection. The results of our study indicate that edible insects play an important role in the transmission of tapeworms to birds, insectivorous animals and humans.

*Pharyngodon* spp. are parasitic nematodes that colonize exotic animals in both wild and captive environments [85, 86]. These parasites are more prevalent in captive pets than in wild animals [85, 86], which could be correlated with edible insects. In our study, insects that had previous contact with animals were significantly more often vectors of *Pharyngodon* spp. our results indicate that insects act as mechanical vectors for the transmission of the parasite’s developmental forms. The role of insects as definitive hosts for *Pharyngodon* spp. has not been confirmed by research. Human infections caused by *Pharyngodon* spp. had been noted in the past [87], but these nematodes are no longer significant risk factors of potential zoonotic disease.

*Physaloptera* spp. form cysts in the host’s hemocoel approximately 27 days after ingestion [88]. Cawthorn and Anderson [89], demonstrated that crickets and cockroaches can act as intermediate hosts for these nematodes. Our study is the first ever report indicating that *Physaloptera* spp. can be transmitted by mealworms and migratory locusts. Insects can act as vectors in the transmission of these parasites, in particular to insectivorous mammals. Despite the above, definitive hosts are not always infected [88, 89]. Cockroaches play an important role in the transmission of the discussed parasites, including zoological gardens [90]. A study of experimentally infected flour beetles (*Tribolium confusum*) demonstrated that Spirurids can also influence insect behavior [91]. Behavioral changes increase the risk of insectivores selecting infected individuals.

Spiruroidea are parasitic nematodes which require invertebrate intermediate hosts, such as dung beetles or cockroaches, to complete their life cycle [92]. In grasshoppers, *Spirura infundibuliformis* reach the infective stage in 11-12 days at ambient temperatures of 20-30°C [93]. Research has demonstrated that these insects are reservoirs of Spiruroidea in the natural environment [94]. These parasites form cysts in insect muscles, hemocoel and Malpighian tubules. They colonize mainly animals, but human infections have also been reported. According to Haruki et al. [95], Spiruroidea can infect humans who accidentally consume intermediate hosts or drink water containing L3 larvae of *Gongylonema* spp. (nematodes of the superfamily Spiruroidea). The prevalence of Spiruroidea in insects has never been studied in Central European insects. In our study, these nematodes were identified mainly in farms importing insects from outside Europe.

Acanthocephala are obligatory endoparasites of the digestive tract in fish, birds and mammals, and their larvae (acanthor, acanthella, cystacanth) are transmitted by invertebrates. The prevalence of these parasites in wild insects has never been studied. In cockroaches, Acanthocephala species such as *Moniliformis dubius* and *Macracanthorhynchus hirudinaceus* penetrate the gut wall and reach the hemocoel [96]. The outer membrane of the acanthor forms microvilli-like protuberances which envelop early-stage larvae [97]. The influence of acanthocephalans on insects physiology has been widely investigated. The presence of *Moniliformis moniliformis* larvae in cockroach hemocoel decreases immune reactivity [98], which, in our opinion, can contribute to secondary infections. Thorny-headed worms influence the concentration of phenoloxidase, an enzyme responsible for melanin synthesis at the injury site and around pathogens in the hemolymph [99, 100]. There are no published studies describing the impact of acanthocephalans on insect behavior. A study of crustaceans demonstrated that the developmental forms of these parasites significantly increased glycogen levels and decreased lipid content in females [101]. Thorn-headed worms also compromise reproductive success in crustaceans [102]. Further research into arthropods is needed to determine the safety of insects as sources of food and feed. Acanthocephalans have been detected in insectivorous reptiles [103], which could indicate that insects can act as vectors for the transmission of parasitic developmental forms.

Pentastomida are endoparasitic arthropods that colonize the respiratory tract and body cavities of both wild and captive reptiles [104]. Pentastomiasis is considered a zoonotic disease, in particular in developing countries [105]. The presence of mites, which resemble pentastomid nymphs during microscopic observations, should be ruled out when diagnosing pentastomiasis in insect farms. The role of insects of intermediate hosts/vectors of pentastomid nymphs has not yet been fully elucidated. However, Winch and Riley [106] found that insects, including ants, are capable of transmitting tongue worms and that cockroaches are refractory to infection with *Raillietiella gigliolii*. Esslinger [107], and Bosch [108], demonstrated that *Raillietiella* spp. rely on insects as intermediate hosts. Our study confirmed the above possibility, but we were unable to identify the factors which make selected insects the preferred intermediate hosts. The choice of intermediate host is probably determined by the parasite species. We were unable to identify pentastomid nymphs to species level due to the absence of detailed morphometric data. Our results and the findings of other authors suggest that insects could be important vectors for the transmission of pentastomids to reptiles and amphibians [106, 109].

### Prevalence

The prevalence of parasitic infections in insects has been investigated mainly in the natural environment. Thyssen et al. [110] found that 58.3% of German cockroaches were carriers of nematodes, including Oxyuridae eggs (36.4%), Ascaridae eggs (28.04%), nematode larvae (4.8%), other nematodes (0.08%) and Toxocaridae eggs (0.08%). Cestoda eggs (3.5%) were also detected in the above study. Chamavit et al. [68] reported the presence of parasites in 54.1% of cockroaches, including *Strongyloides stercoralis* (0.8%), *Ascaris lumbricoides* (0.3%), *Trichuris trichuria* (0.3%), *Taenia* spp. (0.1%), *Cyclospora* spp. (1.3%), *Endolimax nana* (1.3%), *B. hominis* (1.2%), *Isospora belli* (9.6%), *Entamoeba histolytica*/*E. dispar* (4.6%), *Cryptosporidium* spp. (28.1%), *Chilomastix mesnilli* (0.3%), *Entamoeba coli* (4.0%), *Balantidium coli* (5.8%) and *Iodamoeba butschlii* (0.1%). Human-specific parasites such as Oxyuridae, Ascaridae, *Trichuris* spp. and *Taenia* spp. were not detected in our study, which suggests that the analyzed insects did not have access to the feces of infected humans. In a study of wild cockroaches in Iraq, the prevalence of parasitic developmental forms was nearly twice higher (83.33%) than in our study [82]. Iraqi cockroaches carried *E. blatti* (33%), *N. ovalis* (65.3%), *H. diesingi* (83.3%), *Thelastoma bulhoe* (15.4%), *Gordius robustus* (1.3%), *Enterobius vermicularis*, (2%) and *Ascaris lumbricoides* (1.3%). Unlike in our experiment, *H. diesigni* was the predominant nematode species in Iraqi cockroaches. The cited authors did not identify any developmental forms of tapeworms. Tsai and Cahill [111] analyzed New York cockroaches and identified *Nyctotherus* spp. in 22.85% of cases, *Blatticola blattae* in 96.19% of cases, and *Hammerschmidtiella diesingi* in 1.9% of cases. The results of our study suggest that farmed edible insects are less exposed to certain parasites (Ascaridae, *Enterobius* spp.) that are pathogenic for humans and animals. The absence of human-specific nematodes and roundworms could be attributed to the fact that the analyzed farms were closed habitats without access to infectious sources. In the work of Fotedar et al. [112], the prevalence of parasites was determined at 99.4% in hospital cockroaches and at 94.2% in household cockroaches. The percentage of infected cockroaches was much higher than in our study, which could indicate that environmental factors significantly influence the prevalence of selected parasites species. Our observations confirm that the risk of parasitic infections can be substantially minimized when insects are farmed in a closed environment. The high prevalence of selected developmental forms of parasites in the evaluated insect farms could be attributed to low hygiene standards and the absence of preventive treatments. Parasitic fauna in insect farms have never been described in the literature on such scale. A study of cockroaches from the laboratory stock of the Wrocław Institute of Microbiology (Poland) revealed the presence of ciliates in all insects and the presence of nematodes in 87% of insects [113]. These results could be attributed to the fact that all examined insects were obtained from a single stock, which contributed to the re-emergence of parasitic infections. Similar observations were made in several insect farms in the current study.

Edible insect processing like cooking or freezing may inactivate parasitic developmental forms. Tanowitz et al. [114] reported that *Teania solium* is killed by cooking the pork to an internal temperature of 65°C or freezing it at 20°C for at least 12 hours. Smoking, curing or freezing meat may also inactivate protozoa like *Toxoplasma gondii* [115]. The use of microwaves may be ineffective [115]. On the example of *Anisakis simplex*, it has been proven that cooking and freezing can significantly improve food safety in relation to this nematode [116]. Also boiling insect for 5 min is an efficient process for eliminating Enterobacteriaceae [117]. Simple preservation methods such as drying/acidifying without use of a refrigerator were tested and considered promising [117]. However, there is a need of thorough evaluation of insect processing methods, including temperatures and time of cooking / freezing to prevent possible parasitic infections. Despite, food preparation processes parasite allergens may still be detected [116].

Insects may also be a bacterial vector / reservoir, but currently there are no data available for bacteriological tests in breeding insects. It has been proven that insects can be an important epidemiological factor in the transmission of bacterial diseases [3]. One of the most important bacteria that are transmitted by insects include *Campylobacter* spp. [118] and Salmonella spp. [119]. Kobayashi et al. [120] showed that insect may be also a vector of *Escherichia coli* 0157:H7. Free-living cockroaches harbored pathogenic organisms like *Escherichia coli*, *Streptococcus* Group D, *Bacillus* spp., *Klebsiella pneumoniae*, and *Proteus vulgaris* [121]. In vitro studies have shown that some species of insects may also be the reservoir of *Listeria monocytogenes* [122]. In our opinion further research should also focus on the microbiological safety of edible insect breeding.

Due to the fact that the identification of parasites was based on morphological and morphometric methods, further molecular research should focus on the precise determination of individual species of identified parasites in order to determine the real threat to public health. The results of this study indicate that edible insects play an important role in the epidemiology of parasitic diseases in vertebrates. Edible insects act as important vectors for the transmission of parasites to insectivorous pets. Insect farms that do not observe hygiene standards or are established in inappropriate locations (eg. houses) can pose both direct and indirect risks for humans and animals. Therefore, farms supplying edible insects have to be regularly monitored for parasites to guarantee the safety of food and feed sources. Amount of parasites is related to cause the human and animal diseases therefore in the future quantitative studies of parasite intensity in insect farms should be performed. In our opinion, the most reliable method of quantitative research would be Real-Time PCR method. Insect welfare standards and analytical methods should also be developed to minimize production losses and effectively eliminate pathogens from farms.
